# Empowering parents in early motor interventions for infants born preterm: insights from the Norwegian physical therapy study in preterm infants through the lens of a critical interpretative synthesis

**DOI:** 10.3389/fpsyg.2026.1750035

**Published:** 2026-03-23

**Authors:** Marit Sørvoll, Gay L. Girolami, Tordis Ustad, Kari Anne I. Evensen, Suzann K. Campbell, Gunn Kristin Øberg

**Affiliations:** 1 Department of Health and Care Sciences, UiT The Arctic University of Norway, Tromsø, Norway; 2 Department of Pediatric Rehabilitation, The University Hospital North Norway, Tromsø, Norway; 3 Department of Physical Therapy, University of Illinois Chicago, Chicago, IL, United States; 4 Infant Motor Performance Scales, LLC, Chicago, IL, United States; 5 Department of Clinical and Molecular Medicine, Norwegian University of Science and Technology (NTNU), Trondheim, Norway; 6 Clinic of Rehabilitation, St. Olavs Hospital, Trondheim University Hospital, Trondheim, Norway; 7 Department of Rehabilitation Science and Health Technology, Oslo Metropolitan University, Oslo, Norway

**Keywords:** critical interpretative synthesis, enactive theory, NICU (neonatal intensive care unit), NOPPI, parent-administered intervention, pediatric physical therapy, very preterm infants

## Abstract

**Background:**

Infants born very preterm are at risk for motor impairments, highlighting the need for early interventions. The Norwegian Physical therapy study in Preterm Infants (NOPPI) investigated the effects of a parent-administered early motor intervention program in the Neonatal Intensive Care Unit and explored parents’ perceptions of administering the intervention, providing a foundation to explore both the potential and challenges of empowering parents in this role.

**Objective:**

To develop a comprehensive understanding of the depth and complexity of implementing parent-administered interventions.

**Methods:**

We applied a critical synthesis including five quantitative and one qualitative study of the NOPPI. Evidence was analyzed and combined to develop a conceptual framework.

**Results:**

The synthesis highlighted the critical role of active parental involvement and sense-making processes in fostering the motor development of very preterm infants. Using enactive framework as a guiding lens, three interconnected concepts emerged as central to understanding and implementing parent-administered interventions: knowing, doing, and being/becoming. These three dimensions are deeply interwoven and cannot be fully understood in isolation. Together, they form a comprehensive framework that underscores the complexity of parent-administered interventions highlighting knowledge development through embodied interactions, the importance of practical actions and strategies, and the dynamic and evolving identities of parents, infants, and physical therapists.

**Conclusion:**

The critical synthesis of the NOPPI highlights the need for early interventions to consider enactive concepts of knowing, doing, and being/becoming in the planning and implementation of early interventions to optimize outcomes for infants born very preterm and their parents.

## Introduction

Children born very preterm (i.e., <32 weeks gestational age (GA)) are at risk for developmental impairments (Burnett et al., 2025). To positively influence the developmental trajectories of these infants, early intervention paradigms with a preventative focus have been developed (Anderson et al., 2020). Early intervention, defined as interventions initiated within the first year after birth, occurs at a time when the brain of the infant born very preterm is both vulnerable and undergoing significant growth, including development of grey and white matter (Spittle and Treyvaud, 2016; Anderson et al., 2020). While many of these interventions are administered by a range of health-care professionals (Anderson et al., 2020; Baraldi et al., 2020; Edney and McHugh, 2023), the literature highlights the importance of family centered approaches to support the development of very preterm children (Raghupathy et al., 2025b). A key component is the recognition of the critical role that the early parent–child relationship quality plays in shaping developmental outcomes for very preterm children. Strong parent–child relationships significantly influence socioemotional, cognitive, neurobiological, and health outcomes, and underscore the importance including parent-infants dyads in early intervention protocols (Frosch et al., 2021; Raghupathy et al., 2025b).

Therefore, a family-centered approach involves empowering and teaching parents strategies to independently facilitate and enhance early motor behaviors (Raghupathy et al., 2025b) and at older ages to focus on daily activities (Sweeney et al., 2010; Rosenbaum et al., 2014). By engaging early in interactions and activities such as guided touch and sensory-motor input through handling and play, parents are provided with opportunities to strengthen their bond with their infant (Anderson et al., 2020). These early interactions also support the infant’s brain development (Anderson et al., 2020), as brain development is experience-dependent and emerges through the dynamic interaction of genetic, biological, parenting, and environmental factors (Welch et al., 2017). Research shows that early interventions involving both parents and infants are particularly effective, significantly impacting infants’ cognitive and behavioral outcomes (Spittle and Treyvaud, 2016; Anderson et al., 2020), while the impact on motor outcomes is not well-established (Spittle and Treyvaud, 2016).

The design, intensity, duration and delivery of early interventions vary considerably (Spittle and Treyvaud, 2016; Anderson et al., 2020). There is no consensus on the type of intervention, when to begin and how long to intervene (Spittle and Treyvaud, 2016; Anderson et al., 2020). This is particularly true regarding the effects of parent-administered early intervention programs. In addition, while there is a recognition of the importance of parental involvement, the amount, nature, and effectiveness of parental involvement in intervention delivery varies greatly (Spittle and Treyvaud, 2016; Anderson et al., 2020). Furthermore, there is still much to learn about which processes are essential for facilitating effective child and parental learning and development (Benzies et al., 2013; Anderson et al., 2020). Therefore, to gain knowledge about effective early interventions for infants born very preterm, it is essential to identify the key concepts that influence both the implementation and outcomes of these interventions (Benzies et al., 2013). Additionally, understanding how parent participation can be most effectively harnessed is crucial for optimizing parent-infant interactions while addressing infant developmental outcomes (Boynewicz et al., 2024).

To delve deeper into these issues and the complexities of parent-administered interventions, we synthesized findings from the Norwegian Physical Therapy study in Preterm Infants (NOPPI), a longitudinal study in which parents played a significant role in the intervention (Øberg et al., 2012). Conducted between 2010 and 2014, the NOPPI introduced a three-week preventative motor intervention designed to enhance motor development through dynamic social parent-infant interactions, engagement, and handling. Physical therapists (PTs) provided training to parents on how to administer the intervention, and the parents were given the agency to adapt the intervention activities to the individual needs of their infant (Øberg et al., 2012). While the intervention placed significant responsibility on parents, it also highlighted the potential for fostering parental agency and competence in supporting their infant’s development. The overarching aim of the NOPPI was to assess the effects of a parent-administered program in the Neonatal Intensive Care Unit (NICU), as well as the parents’ experiences. By including findings that demonstrate both the effects of the intervention up to two years of corrected age (CA) and the parents’ experiences of implementing the intervention, the study provides valuable insights into the complexities of empowering parents to take an active role in their infant’s intervention program.

This synthesis provides critical insight into processes that underpin successful interventions to foster optimal development for infants at risk and the role of the parent in these interventions. By moving beyond a mere summary of results, we aim to identify, describe and reconceptualize salient overarching key concepts relevant to the effectiveness of the intervention, as well as the strengths and limitations of the approach. These insights can inform the design and implementation of future early intervention programs that rely on active parent involvement. Ultimately, this work seeks to strengthen early intervention practices and improve outcomes for families by advancing knowledge about processes that underpin successful parent-administered interventions.

## Methods

### Study selection

The articles synthesized in this paper are all derived from the NOPPI and represent all published articles synthesizing data from the study. The articles included five quantitative articles employing a pragmatic, multicenter, single-blinded randomized clinical trial design (Ustad et al., 2016; Fjørtoft et al., 2017; Øberg et al., 2020; Ustad et al., 2021; Øberg et al., 2022) and one article utilizing a qualitative interview design (Øberg et al., 2018). The quantitative articles are focused on examining the effects of the intervention on the infants’ motor outcomes at term, 3-, 6-, 12- and 24- months CA, while the qualitative article involved individual interviews with parents to gain a deeper understanding of their perceptions and experiences of administering the early motor intervention program with their infants born prematurely (see Table 1).

**Table 1:** Overview of the articles in the NOPPI.

Title	Authors/year	Aim/purpose	Study design	Participants assessed	Intervention	Outcome measures	Key findings	Conclusion
Early Parent-Administered Physical Therapy for Preterm Infants	Ustad et al. (2016)	Investigate short-term effects of parent-administered PT on motor performance	RCT	135 infants, ≤32 weeks GA	Parent-administered physiotherapy in NICU	TIMPSI TIMP	Improved motor performance at 37 weeks PMA	Short-term benefits observed
Does a Parent-Administered Early Motor Intervention Influence General Movements	Fjørtoft et al. (2017)	Examine effects on fidgety movements at 3 months	RCT	130 infants, ≤32 weeks GA	Parent-administered intervention	GMA AMR	No significant difference in fidgety movements or AMR	Early intervention did not alter GMs or AMR
Parents’ Perceptions of Administering a Motor Intervention	Øberg et al. (2018)	Explore parents’ perceptions of administering intervention	Qualitative	7 parents of 8 infants, ≤32 weeks GA	Parent-administered intervention	Interviews N = 11	Positive impact on bonding and empowerment	Enhanced parent-infant interaction
Effects of a Parent-Administered Exercise Program in the NICU	Øberg et al. (2020)	Examine effectiveness and dosing impact on motor outcomes	RCT	138 infants, ≤32 weeks GA	Parent-administered exercise program	TIMPSI TIMP	No difference at 3 months, dose mattered	Dose positively associated with outcomes
General Movement Optimality Score and Trajectories	Ustad et al. (2021)	Present detailed GM scores and trajectories	Retrospective	141 infants, ≤32 weeks GA	Parent-administered physiotherapy	GMA AMR	No significant changes in GMs or AMR	Early intervention did not alter GMs or AMR
Two-Year Motor Outcomes and Dose	Øberg et al. (2022)	Examine 24-month motor outcomes and dosing effects	RCT	127 infants, ≤32 weeks GA	Parent-administered intervention	TIMPSI TIMP PDMS-2	No difference at 24 months, dose mattered	Dose positively associated with outcomes

NICU, Neonatal intensive Care Unit; PDMS-2, Peabody Developmental Motor Scales, Second Edition; TIMPSI, Test of Infant Motor Performance Screening Items; GMA, General Movement Assessment; TIMP, Test of Infant Motor Performance; GA, Gestational Age at birth; AMR, Assessment of Motor Repertoire; PMA, Post Menstrual Age.

### A critical interpretative synthesis

Given the diverse methodological approaches in the NOPPI, we adopted a critical interpretative synthesis framework (Dixon-Woods et al., 2005; Aveyard et al., 2021) for this study. This framework allowed us to systematically combine evidence from both the quantitative studies and the qualitative study in relation to the implemented intervention, enabling a deeper understanding of the dynamics and outcomes of a parent-administered early intervention program. Critical interpretive synthesis involves both inductive and interpretative processes, with its primary aim being the development and integration of significant concepts and theories (Dixon-Woods et al., 2005; Aveyard et al., 2021).

To operationalize this critical interpretative synthesis framework, we employed three strategies of interpretive synthesis in our analysis (Thorne et al., 2004; Dixon-Woods et al., 2005). First, we conducted a textual narrative synthesis summarizing study characteristics (e.g., aims, designs, methods, and main findings) in a table to identify commonalities and differences among the studies, focusing on the intervention in relation to key findings and conclusion (Table 1). This process was completed by two of the authors (MS and GKØ), who noted largely similar observations with only minimal discrepancies. Any differences were resolved by returning to the original articles to review and come to a consensus regarding the data to be included in the table.

Secondly, we compared all the main points from each article to ensure that key findings from all six articles were included in the analyses (Table 2). For our project, we deliberately chose not to specify the use of theoretical concepts in advance to categorize the data. Instead, one of our aims was to allow the themes to emerge inductively from our analysis of the evidence as we systematically appraised the data relative to the evolving themes. The analysis was completed by three of the authors (MS, GLG, and GKØ). Each author had read the articles independently and was thoroughly familiar with their content. This was followed by a collaborative discussion process to ensure that the central findings from each study were incorporated, and to reflect on and identify preliminary concepts appropriate for the aims of the present synthesis. This process involved moving back and forth between the individual parts and the whole, meaning that the themes were continuously evaluated and adjusted in relation to each other and the overall context of the intervention and its outcomes. After initial themes were identified and possible interpretations considered, we then again reviewed the articles and engaged with theory to fine-tune and validate these themes. The evolving themes strongly resonated with key principles in enactive theory, which emphasizes cognition as emerging through dynamic interactions between individuals and their environment (Fuchs and De Jaegher, 2009). We therefore found that this theoretical perspective provided a valuable lens for understanding the findings and the relationships among them in our further analysis, thereby deepening and enriching our interpretations.

**Table 2:** Worksheet for analysis and synthesis.

Intervention characteristics	Study	Findings	Our translation	Relationship pattern	Concepts
Early parent-administered intervention through 34-,35-, and 36-weeks postmenstrual age, conducted twice daily. The intervention educated parents in individualized handling and motor stimulation to improve postural control, head control, and midline orientation in the infant. The parent-led exercises were integrated with communication and social interaction between parent and infant.	Ustad et al. (2016)	The intervention group showed a significant improvement in motor performance at 37 weeks postmenstrual age compared to the control group, with an estimated difference in z scores of 0.42 and an effect size of 0.40. The intervention was well-tolerated by the infants, with no adverse events reported.	The immediate benefits of the intervention are influenced by mutual engagement, where the infant demonstrates competence, and parents gain confidence to challenge their infant through active participation in the intervention. Importance of parental involvement: Interaction Mutual engagement Embodied action	Dynamic and reciprocal embodied processes unfolding between parents and infants through doing and knowing-that and knowing-how. Being/becoming as sense-making processes.	Knowing Doing Being/becoming
	Fjørtoft et al. (2017)	No significant difference was found between the intervention and control groups regarding fidgety movements or movement character at 3 months corrected age (CA). Approximately half of the infants in both groups exhibited an abnormal movement character.	The intervention cannot influence fidgety movements or overall movement character, supporting the notion that these are influenced by brain injury and are good predictors of later neurological impairments.	Interaction and embodied doing do not alter final outcomes. There is a need for further research on the timing of interventions in relation to their impact on General Movements.	
	Øberg et al. (2018)	Parents reported a significant positive impact on bonding with their infants and an increased sense of empowerment and competency. The intervention helped parents overcome initial fears of handling their infants, enhancing their awareness of the infants’ responses and capabilities. Parents felt more in control of decision-making and more interactive with their infants, which reinforced attachment and contributed to their perception of their infants as competent and independent beings. Overall, the parent-administered intervention program was perceived as beneficial in fostering parent-infant bonding and enhancing parental confidence and empowerment.	Importance of parental support, embodied action, and different forms of knowledge. Interaction Meaning-making Mutal engagement Empowerment Confidence	Dynamic and reciprocal embodied processes unfolding between parents and infants through doing and knowing-that and knowing-how. Being/becoming as sense-making processes.	
	Øberg et al. (2020)	No significant difference in motor performance (TIMP z-score) was found between the intervention and control groups at 3 months CA. A significant positive relationship was observed between the total intervention dose and improved motor outcomes, indicating that higher intervention doses were associated with better motor performance. The odds of having a clinical z-score <0 at 3 months CA were about six times higher for infants with less than the median intervention time compared to those with longer intervention time.	Need for closer follow-up and support for parents to ensure they develop the skills and confidence necessary to effectively manage and implement the intervention: Dosage Frequency and duration Adherence to intervention Support and guidance to perform embodied actions To empower parents Interaction Flexibility Meaning-making Knowing	Dynamic and reciprocal embodied processes unfolding between parents and infants through doing and knowing-that and knowing-how. Being/becoming as sense-making processes.	
	Ustad et al. (2021)	Both groups showed a high proportion of abnormal General Movements at 34- and 36-weeks postmenstrual age. No significant differences were found between the intervention and control groups in terms of general movement optimality scores (GMOS) or motor repertoire at 3 months post term age.	The intervention cannot influence fidgety movements or overall movement character, supporting the notion that these are influenced by brain injury and are good predictors of later neurological impairments	Interaction and embodied doing do not alter final outcomes. There is a need for further research on the timing of interventions in relation to their impact on General Movements.	
	Øberg et al. (2022)	No significant difference in motor performance was found between the intervention and control groups at 24 months CA using the Peabody Developmental Motor Scales-2 (PDMS-2). A significant positive association was found between the intervention dose and improved Gross Motor and Total Motor scores on the PDMS-2. Longitudinal analysis revealed initial improvement in both groups, followed by a decline in motor function between 6- and 24-months CA relative to age norms.	Importance of sustained intervention: Duration Dosage Knowing Adherence Support and guidance to perform embodied actions To empower parents	Dynamic and reciprocal embodied processes unfolding between parents and infants through doing and knowing-that and knowing-how. Being/becoming as sense-making processes.	

Finally, we synthesized interpretations from across all the articles to generate a comprehensive synthesis within a coherent framework (Dixon-Woods et al., 2005) (Table 2). This process was also conducted by MS, GLG, and GKØ. During this stage, we utilized handwritten maps and different color codes to organize and analyze the data, which allowed us to develop an integrated understanding of the findings. This process accentuated the dynamic and reciprocal processes unfolding between parents and infants in relation to motor development. Connecting theory to the findings provided new perspectives for further developing our synthesizing argument. Grounded in the aggregated evidence and our interpretation, this approach allowed us to unify several aspects of early parent-administered intervention (NOPPI) into a more coherent, descriptive, and advantageous framework, leading to the generation of synthetic constructs. Through our critical analysis, synthesis, and application of the enactive theoretical perspective, a network of interrelated insights emerged concerning the contents and enactment of parent-administered early intervention.

### The research team

Because critical interpretive synthesis requires ongoing reflexive engagement with how researchers’ backgrounds, assumptions, and interpretive lenses shape the analytic process (Aveyard et al., 2021), it was important to consider the composition of our research team and the perspectives each member brought to the synthesis. Our research team is comprised of scholars with diverse clinical physical therapy backgrounds and a wide range of research experience which contributed to the strength and reflexive rigor of the synthesis. Four members of the team are from Norway (MS, TU, KAIE, and GKØ) and two from the U. S. (GLG and SKC). This diversity of perspectives enabled us to question assumptions, challenge interpretive biases, and ensure that the emerging concepts were grounded in a broad understanding of parent-administered early interventions in both the Norwegian and U. S healthcare systems. Furthermore, four of the authors (GKØ, TU, GLG, and SKC) were involved in the conceptualization and implementation of the NOPPI, contributing detailed knowledge to the design, intentions, and implementation of the study. In contrast, the remaining two authors (MS and KAIE) joined the project after the intervention was completed. This combination of perspectives brought both in-depth familiarity with the study and an external viewpoint to the critical interpretive synthesis, enriching the process by introducing fresh insights and fostering a more nuanced and reflexive analysis.

## Results

Through our critical interpretative synthesis and application of the enactive theoretical perspective, a network of interrelated insights emerged concerning the contents and enactment of parent-administered early interventions. In the following, we first provide a contextual background of the NOPPI, followed by a synopsis of its findings, and finally present our translation and synthetic construct.

### The NOPPI – a contextual background

#### The NOPPI sample

The NOPPI study sample consisted of 153 very preterm infants who were recruited from three Norwegian hospitals with level III NICUs. Infants were eligible for inclusion if they were medically stable and able to tolerate handling, and if their parents understood and spoke Norwegian. Exclusion criteria were triplets or higher-order multiples, congenital malformations, genetic syndromes, and infants who had undergone major surgery. Parents also had to consent to participate in follow-up assessments. Randomization was conducted using a web-based system developed and managed by the Unit of Applied Clinical Research at the Norwegian University of Science and Technology. Allocation was stratified by GA at birth (<28 + 0 weeks vs. >28 weeks) and by recruitment site. Twins were placed in the same group because the nature of the intervention made it impossible to blind group assignment from parents and the PT delivering the training. Outcome measurement tools were the *Test of Infant Motor Performance Screening Items* (TIMPSI) (at 34 weeks postmenstrual age (PMA)), *Test of Infant Motor Performance* (TIMP) (at 37 weeks PMA and 3 months CA), *General Movement Assessment (GMA)* (at 34 and 36 weeks PMA and 3 months CA), *Assessment of Motor Repertoire at 3 to 5 Months* (AMR) (at 3 months CA), and *Peabody Developmental Motor Scales–2* (PDMS-2) (at 6, 12, and 24 months CA) (Øberg et al., 2012). Of the 217 eligible infants, parents of 153 infants agreed to participate, resulting in a participation rate at baseline of 70.5%. At 24 months CA, 127 participants attended follow-up assessments, corresponding to a follow-up rate of 84.3%. However, because some infants did not attend all follow-up visits, the number of infants assessed varies across the quantitative articles (see Table 1).

A subsample of parents of eight infants, including one pair of twins, from the intervention group were invited for qualitative interviews using purposive sampling to ensure maximum variation in relevant characteristics. This approach aimed at capturing a broad range of parental experiences with respect to administering the intervention. The selected infants represented diversity in sex, GA at birth (24–32 weeks), birthweight (650–1985 g), medical history, number of siblings, and family structure, as well as variation in parental education (Øberg et al., 2018).

#### The NOPPI intervention

The NOPPI intervention (Øberg et al., 2012) integrates insights from multiple sources, combining key elements from the modified version of the “Mother-Infant Transaction Program”, as studied by Kaaresen et al. (2008) alongside components from Girolami’s handling and motor stimulation program (neurodevelopmental therapy) that have demonstrated positive effects on the motor development of infants born preterm (Girolami and Campbell, 1994). This comprehensive approach is rooted in the understanding that motor skills are deeply embedded within social interactions, emphasizing the ability to read and interpret the child’s bodily expressions.

The parent-administered exercise program, conducted from 34 through 36 weeks PMA for 10 min twice daily, was designed to enhance postural and motor control of the head and trunk, as well as midline orientation of the head, arms, and legs across four positions: prone, supine, side-lying, and supported sitting. Further, Girolami’s protocol was modified to actively involve the infant in transitions from supine to side-lying and from side-lying to sitting, promoting active participation and motor skill development (Øberg et al., 2012). In each position, guided movements and intermittent manual compressions were applied to relevant muscle groups and joints providing tactile, vestibular, and proprioceptive inputs, paired with visual and auditory inputs. The activities were designed to improve body awareness, increase isometric coactivation, and enhance the ability to initiate and sustain muscle activity for functional performance. The initial starting point for the intervention was tailored by the PT based on the infant’s performance on the TIMPSI (Campbell et al., 2008) and their tolerance for movement. At least one activity in each of the four positions and one transition activity was always included.

The protocol was further adapted from a therapist-delivered to a parent-delivered approach (Øberg et al., 2012). Experienced pediatric PTs trained parents to skillfully execute the intervention strategies, ensuring they were comfortable with the process. Parents were taught to observe and interpret their infant’s behaviors and physiological signs of pleasure and stress. PTs emphasized the importance of communication and social interaction between parent and infant, advising that the infant should be in State three (drowsy or semi-dozing) or State four (alert with a bright look), according to Prechtl’s scale of state (Prechtl, 1977). Parents were instructed to pause the intervention to calm the infant if stress signs such as grimacing, changes in skin color, irregular respiration, or loss of muscle tone were observed. Parents were given autonomy to adjust the time of the session and the session length based on their infant’s behavior or physiological condition. Additionally, parents were asked to provide a daily summary of the number of sessions and the duration (in minutes) of each session.

A minimum of three sessions with the PT were provided to teach, supervise, and support parent learning. Additionally, parents could contact the PT for further support or clarification regarding the exercises. The intervention protocol was documented in a “Play-Book” with photos and written instructions, which was provided to each parent in the treatment group.

Infants in the standard care group were provided with routine nursing care based on the principles outlined in the Newborn Individualized Developmental Care and Assessment Program (NIDCAP) (Als, 2009).

### The NOPPI findings – a synopsis

The quantitative studies of the NOPPI sought to assess the effects of a parent-administered intervention where the endpoint was motor development at 24 months CA (Fjørtoft et al., 2017; Øberg et al., 2020; Ustad et al., 2021; Øberg et al., 2022) (Table 1). Upon discharge from the NICU, motor outcomes favored the intervention group, showing an effect size of 0.4 (Ustad et al., 2016). No intervention was provided after hospital discharge, but motor performance continued to be assessed. By three months CA, the difference in motor performance between groups was no longer evident (Øberg et al., 2020).

Adherence to the intervention program in the NICU was assessed through parental documentation of session frequency and duration. The average and median total time recorded over the three-week intervention period was 222 min, equating to approximately half of the recommended 420 min (i.e., 10 min x 2 per day). The parents reported that the primary reason for not completing the intervention as intended was the infants’ behavioral states, such as sleepiness, fatigue, hunger, or illness (Øberg et al., 2020). Interestingly, exploratory data analyses revealed that infants who received at least 50% of the recommended intervention dosage, regardless of medical complexity, exhibited significantly better motor outcomes on the TIMP at three months CA (Øberg et al., 2020). At 24 months CA, there was still no difference between the groups on the Peabody Scales; however, a significant positive association remained between the intervention dosage and motor development (Øberg et al., 2022). Moreover, the NOPPI demonstrated that neither the overall motor repertoire nor the General Movements (GMs) before term (i.e., at 36 weeks PMA) and at three months CA, both of which are predictive of later neurological impairments such as cerebral palsy, were influenced by the intervention provided between 34 to 37 weeks PMA, as these characteristics are primarily determined by permanent brain injury (Fjørtoft et al., 2017; Ustad et al., 2021).

The qualitative study explored parents’ experiences of administering the NOPPI intervention with their preterm infants in the NICU (Øberg et al., 2018) (Table 1). The findings revealed the intervention had a significant positive impact on parent-infant bonding, parental confidence, and perceptions of infant competency. Parents reported increased confidence in handling their infants, a deeper understanding of their infants’ needs and abilities, and a strengthened sense of attachment. The intervention helped reduce feelings of helplessness and fear, empowering parents to take an active role in their infants’ care and fostering meaningful parent-infant interactions, autonomy, and decision-making.

### The NOPPI findings – translation and synthetic construct

Drawing on the findings from the six NOPPI articles, we sought to explore the interplay between early intervention practice and motor outcomes in infants born very preterm up to two years of CA. A key element of the NOPPI intervention was individualized handling and motor stimulation integrated into communication and social interaction between the parent and the infant, ensuring that both the parent and the child were active participants (Øberg et al., 2012). This involvement was not only about parent–child interaction but also encompassed knowledge facilitated by the PT’s guidance and training in the three scheduled encounters. Such dynamic, embodied, and interactive parental involvement suggests viewing the intervention as co-constructed embodied processes between parents, infants and therapists. Through our analysis, it became evident that the findings revolved around interaction, mutual engagement, meaning-making, learning, different forms of knowledge, and embodied action in relation to motor development (Table 2). Embodiment refers to the idea that our ability to perceive, understand, and act in the world is fundamentally shaped by our mind–body entities and our ongoing interactions with the environment (Fuchs and De Jaegher, 2009). Our interest was particularly driven by parents’ perceptions of parent-infant bonding, empowerment, and competence, as well as the processes underlying the NOPPI intervention. The findings suggested a potential relationship between parental engagement, specifically the time dedicated to the intervention, and developmental outcomes in the child, as well as the parents’ perceptions of the resilience of their infant (Øberg et al., 2022).

With a specific focus on the relationship between active involvement, sense-making processes, knowing, and motor development, the parents’ ‘active embodied doing’ appeared to be a key to their motivation, engagement, personal growth, and adherence to the program (Øberg et al., 2018). Of particular importance was how knowledge emerged through their doing, empowering their agency and transforming their perceptions of both their infants and themselves, while also fostering reciprocity in the parent-infant and parent-PT interactions. Sense-making therefore seem to be an essential aspect of early parent-administered interventions as it emerges through the dynamic interplay, in which meaning is co-created through shared embodied experiences (De Jaegher and Di Paolo, 2007).

**Figure 1:**
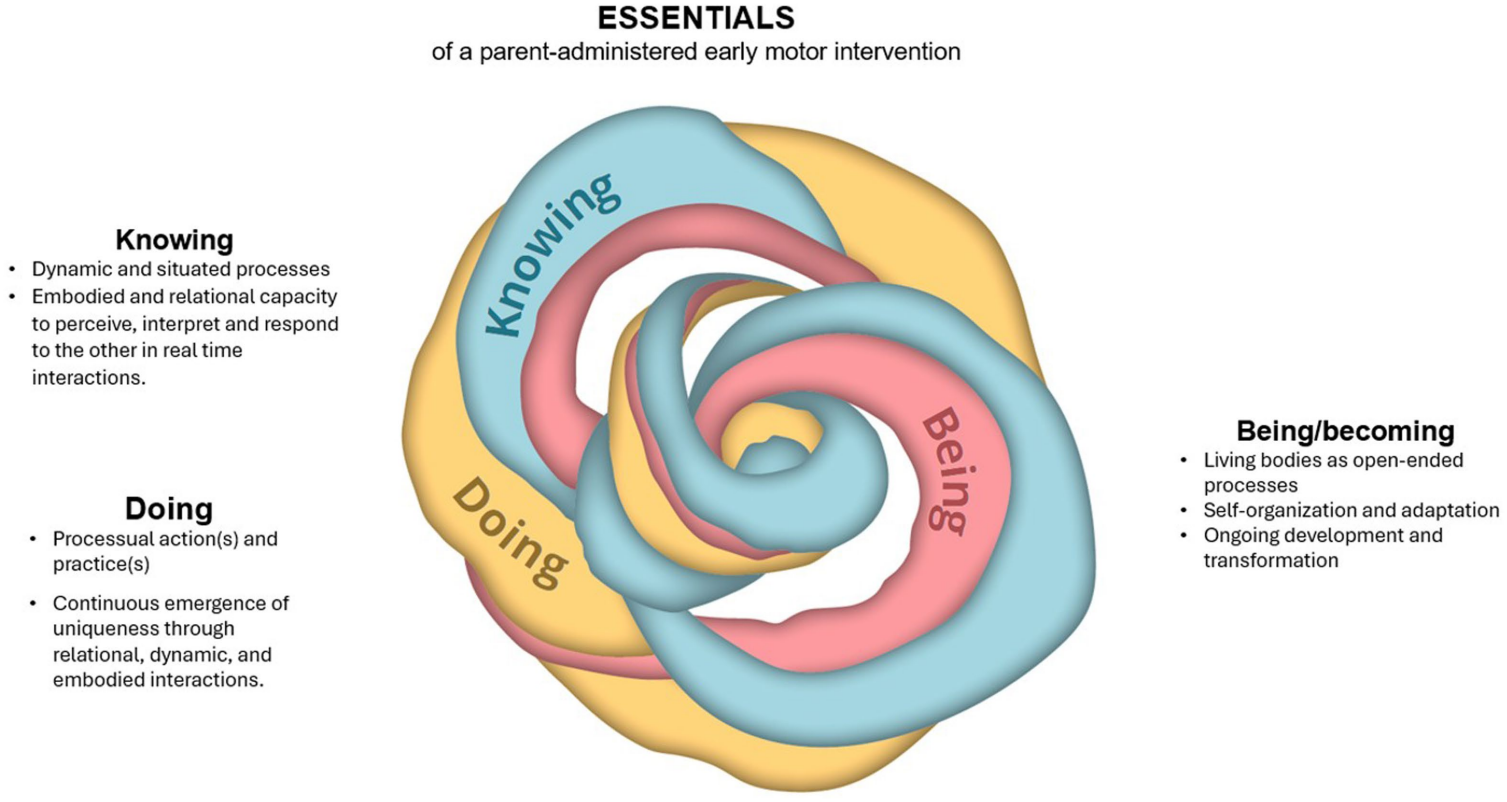
Essentials of early parent-administered interventions.

The prominent features of participation in the early parent-administered intervention were closely linked to how knowledge becomes meaningful when applied through actions, shaping parents’ evolving sense of identity and competence as caregivers. Certain aspects of the implementation of the intervention appeared particularly significant for the child’s motor development. By exploring how these aspects interconnected, we arrived at three deeply intertwined enactive concepts (Di Paolo and De Jaegher, 2022) that encapsulate the complexity embedded within our findings and may hold relevance for other early parent-administered interventions: (1) *knowing*: the understanding and insights gained through interactions between parent, preterm infant, and PT; (2) *doing*: the actions and practices that parents and PTs engage in to support the infant’s development; and (3) *being/becoming*: the existence and identities of the parents, the infant, and the PT as they navigate challenges and opportunities during encounters (Figure 1). Although it is challenging to separate these concepts from one another (Di Paolo and De Jaegher, 2022), we will now briefly introduce them individually in the context of the NOPPI.

### Knowing

Within the framework of enactive theory, knowing emerges as a dynamic and situated process where sense-making, understanding, and insights are gained through interactions with others, also conceptualized as participatory sense-making (De Jaegher and Di Paolo, 2007; Di Paolo and De Jaegher, 2022). This concept of knowing highlights the importance of parents learning through their interactions with their infants and PT, and is not limited to acquiring factual information (knowing-that) but extends to an embodied and relational understanding rooted in actions and experiences (knowing-how) (Di Paolo et al., 2018). Therefore, knowing is not merely technical or procedural knowledge, but an active, ongoing engagement shaped by the dynamic embodied interplay between parent and infant. This interplay naturally incorporates the dimension of doing, as knowing and the acquisition of knowing-how are inseparable from active, embodied engagement and relational handling. Hence, knowing is enacted in the very dynamics of doing.

### Doing

If knowing-how is manifested through actions that involve the practical application of knowledge and skills as a means to foster the infant’s sensorimotor growth and the parents’ intervention competency, then doing represents the active implementation of the intervention as well as the degree to which parents engage independently with their infant. In this context, intervention increasingly becomes the pre-reflected way the parents navigate their doings through processes of skilled (inter)actions. Therefore, doing is not a reaction to external stimuli but ongoing processes of adaptive sense-making (Di Paolo and De Jaegher, 2022), where parents actively can regulate their relation to the infant and environment in ways that sustain their identity. Encompassing the knowing parents gain through their active engagement in doing and the infant’s actions and learning processes, doing and knowing are not isolated phenomena but are deeply embedded in the continuous and dynamic processes of being/becoming, whereby the parents and the infants evolve through their shared interactions.

### Being/becoming

Being/becoming can be understood as the existence and identities of individuals, which is closely related to the enactive concept of autonomy (Di Paolo and De Jaegher, 2022). Autonomy refers to an individual’s capacity for self-organization and adaptation, sustained and supported through a network of interdependent processes that work together to preserve the individual functioning and identity (Di Paolo, 2021). For infants born very preterm, their being is characterized by fragile autonomy. In early interventions, these infants are at a critical stage where their sensorimotor, physiological, and cognitive systems are still developing, making their developmental processes inherently precarious. This notion of precariousness is central to understanding the challenges faced by infants born very preterm, as it reflects the inherent fragility of both the infant and interactional processes with the parents. These processes require continuous effort to maintain individual and interactional organization (De Jaegher and Di Paolo, 2007; Beer and Di Paolo, 2023). In this light, the infant can be seen as a living, sense-making being that encounters a range of structured developmental challenges as they adapt to their new environment outside the womb, which encompasses both physical and social worlds (Sørvoll et al., 2022). Becoming, therefore, refers to the ongoing developmental transformation shaped by embodied interaction and adaptation over time.

This process of being and becoming also extends to the parents. In the NICU, parents often perceive infants as fragile (Raghupathy et al., 2025a). By actively participating in parent-infant interactions, parents may gain confidence and see their child as capable, shifting their perception from one of vulnerability to competence. This shift allows parents to develop a deeper understanding of their infant’s needs and abilities. Such transformative processes underscore the importance of how early interventions are structured and organized, as they not only shape the infant’s development but also influence the parents’ being and becoming. These relational and embodied processes illustrate how early parent-administered interventions can support not only the infant’s developmental outcomes but also the evolving identities of both parents and infants.

## Discussion

The central findings of this critical synthesis, based on our analysis of the six NOPPI article short- and long-term results, indicate that the three concepts of knowing, doing, and being/becoming are essential for the success of early parent-administered interventions for infants born very preterm. These concepts are interwoven, co-defining and influencing one another (Di Paolo and De Jaegher, 2022). For parents to be effectively involved in early interventions, their knowing must always be situated within the context of their personal growth of being/becoming and doing. Similarly, their actions (doing) are both informed by and contribute to their being/becoming and knowing. Simultaneously, the parents’ knowing, doing, and being/becoming also play a critical role in shaping the infant’s own process of being/becoming.

To further understand and elaborate on this, we will critically explore the dynamic interplay of these concepts in relation to the translated NOPPI findings in the discussion.

### The dynamic interplay of knowing, doing and being/becoming

Drawing on the synthesis of the NOPPI findings, early parent-administered interventions appear to be inherently complex because of the interplay and social interaction among the parents, the infant, and the therapist. This complexity arises from the integration of professional knowing-how required to implement the intervention successfully, alongside the intricate interplay between the parent, the infant, and the PT, all within the context of broader social and interactional dynamics. In parent-administered interventions, a significant level of responsibility is placed on parents, who are often in a highly vulnerable situation following the birth of a very preterm infant. This duality makes the administration of the intervention both challenging and rewarding (Øberg et al., 2018; Øberg et al., 2023). Parents frequently face multifaceted challenges, including uncertainty, stress, fear, anxiety, and even depression, with these stressors potentially persisting over time (Ionio et al., 2016; Lean et al., 2018). This underscores the need for careful consideration of potential contributions of parent-administered interventions.

**Figure 2:**
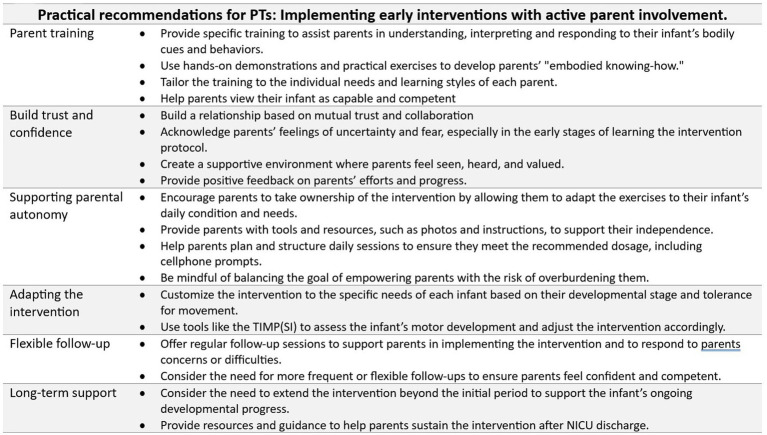
Practical recommendations.

Findings of the NOPPI intervention revealed that, on the one hand, such interventions may foster parental competence (knowing-how), strengthen the parent–child bond (being/becoming), and ensure that interventions are tailored to the child’s unique needs (doing) (Øberg et al., 2018; Øberg et al., 2023). On the other hand, parental difficulties with completing the prescribed dosage (knowing-how and doing) were a critical finding in the NOPPI, as there appeared to be a significant positive association between the amount of intervention delivered and motor outcomes (Øberg et al., 2020; Øberg et al., 2022). These types of challenges were not reported in the Girolami and Campbell’s study (1994), in which the PT administered all aspects of a higher-dosage intervention without reported negative effects on the infants physiological stability and weight gain. Although parents in the NOPPI attributed lower dosage to their infant’s behavioral state, it is plausible that emotional demands, limited supervision, or insufficient ongoing support also contributed. According to the NOPPI findings, placing the responsibility on parents to request additional support or guidance may have made it challenging for them to access the help they needed. This might have limited their opportunity to fully develop the necessary knowing-how to implement the intervention confidently. Therefore, it is plausible that planning for additional support from the PT can address the parents’ understanding about how to assess the readiness of their infant to participate in the intervention. This might include more frequent touchpoints, individualized coaching, and practical guidance on adapting the intervention to the infant’s behavioral state and to sustain a sensitive and competent practice (Figure 2). For example, parents might benefit from learning to identify optimal engagement windows for their infant or establishing consistent daily routines to help entrain the infant’s responses. Such strategies could support parents in navigating the emotional and practical demands of intervention delivery, ultimately improving adherence and outcomes (Figure 2).

Building on the importance of fostering parental competence and sustaining developmental progress, it is crucial to consider the timing, dosing, and duration of early parent-administered interventions. This three-week intervention, conducted before term age, demonstrated a significant effect immediately following its completion (Ustad et al., 2016), providing a positive start for the infants’ motor development, increased parental competence, and parent-infant bonding. In this sense, early parent-administered interventions in the NICU can be considered useful. Furthermore, the relationship between intervention dosage and motor outcomes at 24 months CA in the NOPPI suggests that early parent-administered interventions may affect later motor development when provided with sufficient dosing. However, these findings call for prospective studies to determine the optimal timing and dosing in early parent-administered interventions. It is important to acknowledge that such a short duration is likely insufficient to support the infant’s long-term motor development during childhood. Research indicates that the effects on child development are greater when interventions begin in the NICU and continue after discharge from the hospital (Spittle and Treyvaud, 2016). This highlights the need to plan early parent-administered interventions that provide a strong initial start for the infant but also extend beyond the NICU and are continuously adapted to the child’s ongoing developmental needs.

Another consideration regarding the usefulness of early interventions noted in the research, is the role of skilled PTs, whose embodied knowing-how enables them to take an active role in performing all or part of the intervention, leading to improved motor outcomes for the infant (Girolami and Campbell, 1994; Lee et al., 2010; Lee, 2017; Boynewicz et al., 2024; Balci et al., 2025). These findings underscore the critical importance of PTs’ embodied knowing-how in early interventions, particularly in parent-administered therapy protocols to enhance the parents’ competence (knowing-how and doing). Such evidence suggests that a more effective strategy to optimize the outcomes of future interventions should include a collaborative approach, combining parent-administered and therapist-administered interventions.

However, despite this, it is essential to enable parents to be actively involved and engaged, as research shows that active parental involvement is crucial for the parent–infant relationship and for supporting child development over time (Frosch et al., 2021). The importance of empowering parents through training and fostering their ability to interpret and respond to their infant’s cues through hands-on practice, reflects the integration of knowing and doing. As ethical considerations are intrinsically woven into these transformative practices (Di Paolo and De Jaegher, 2022), being/becoming confident parents requires the ability to navigate the vulnerability and precariousness of their situation while fostering a sense of their autonomy with guidance and support from the PT.

We hypothesize that the intimate connectedness between precariousness and autonomy may help explain why parents in the NOPPI reported feeling empowered to make independent decisions and more confident in engaging interactively with their infants (Øberg et al., 2018). Parents bring diverse experiences, ways of knowing, and coping strategies to the intervention, highlighting the importance of providing individualized support tailored to each parent’s specific needs as they learn to interact with their infant (Figure 2). Developing embodied knowing-how often takes time, as it involves enabling parents to adequately interpret and respond to their infant’s bodily communications and physiological cues, fostering appropriate and responsive caregiving handling and practices. Such competence is essential for supporting the infant’s being/becoming. Taking the infant’s perspective, self-initiated movements support emerging neurological and motor structures, as well as early forms of communication, demonstrating that the infant actively shapes and contributes to the unfolding interaction processes (Sørvoll et al., 2022). This interplay between the infant’s active contributions and the parents’ developing competence underscores the relational and dynamic nature of early parent-administered interventions, where both parents and infants influence and shape each other’s development (being/becoming).

When implementing early parent-administered interventions, it seems crucial to ensure that parents are given the opportunity to actively engage in doing. Taking responsibility, acting independently, and making autonomous decisions may contribute to gaining an understanding of oneself as a parent of an infant born preterm (being/becoming). This indicate the need to start parents’ education earlier to schedule practice sessions, for example by using a doll or a term infant to acquire the psychomotor skills (the knowing-how and doing), thereby allowing them to later focus more on the being and becoming aspects. Such active engagement allows them to develop a deeper sense of empowerment and confidence in their role as a caregiver.

The prominence of the three intertwined enactive concepts, knowing, doing, and being/becoming, may shift depending on the continuously changing needs of the infant-parent dyad. To be clear then, knowing and doing may take a central role when parents are learning to interpret and respond to their infant’s cues, while being/becoming remain present but less pronounced. Similarly, doing may dominate during the practical application of intervention strategies, with knowing and being/becoming supporting the process. In other cases, being/becoming may emerge as the most prominent concept, reflecting the evolving identities and relationships of parents, infants, and PTs, while knowing and doing play more subtle roles (Figure 1). Based on our findings, we propose several practical strategies to enhance intervention outcomes (see Figure 2).

### Methodological considerations

The aim of this critical synthesis has been to develop a deeper understanding of early parent-administered interventions by analyzing the findings from the NOPPI. The strength of this synthesis is that all published articles from the NOPPI study were included and highly relevant for generating new insights and informing the development of concepts. Throughout the process, we engaged in an iterative approach, critically examining underlying assumptions and any contradictions we could identify across the six articles. This allowed us to provide a synthesized analysis of the study findings. In line with recommendations in the research literature (e.g., Aveyard et al., 2021), we also were cognizant of the need to provide transparent account of our analytical process to ensure credibility and rigor.

Another strength is that we actively employed theory to enable generalization of the synthesis findings beyond the NOPPI context. Two key concepts underpinned this process (Aveyard et al., 2021): (1) Theorization and concept formation provided the foundation for analytical generalization, allowing us to extend insights beyond the immediate context of the study. (2) Recognition, which is grounded in the readers’ familiarity with the field or their ability to identify general patterns and characteristics of the phenomenon under study. This familiarity may serve as an additional basis for generalization, as it enables both the authors and the readers to connect the findings to similar contexts or interventions. By employing this methodological approach, we were able to strengthen the relevance of our findings and contribute to the broader understanding of early parent-administered interventions, making them applicable to other cultural contexts, disciplines, or NICU settings outside of Norway.

However, we acknowledge that the limited number of articles (n = 6) may be considered a limitation, as our recommendations for future early parent-administered interventions are solely based on the synthesis of findings from the NOPPI. Another potential limitation of this synthesis is that a different research team analyzing the same set of papers might have arrived at a different theoretical concept. This highlights the subjective nature of critical interpretive synthesis (Aveyard et al., 2021), where the researchers’ backgrounds, perspectives, and analytical approaches can influence the development of concepts and interpretations.

### Final remarks

Based on the results of the NOPPI, the evidence suggests that embodied knowing, doing, being/becoming are very important aspects to consider when designing, planning and implementing early intervention programs using a model of direct parent involvement to foster motor development in the infant born very preterm. This integration appears to be essential for both the child’s short- and long-term development and the parents’ empowerment, as they grow into their role as parents of these infants.

A crucial implication of these findings is the recognition that therapeutic competence cannot be reduced to knowing-that. Rather, it rests in the embodied knowing-how: the practical, relational, and sensorimotor capacity to perceive, interpret, and respond to the infant’s bodily expressions and movement behaviors in real-time interactions. This form of knowing-how is enacted through the PT’s own bodily engagement, including the use of hands-on techniques and movement analysis, which enables the PT to guide and support the parent-infant dyads effectively. Such embodied expertise is essential for translating developmental knowledge into meaningful, adaptive interventions. An important consideration is, however, whether PTs should take a more active role in delivering the interventions in addition to the parents.

Moreover, the enactive approach highlights the significance of social contexts in shaping human development and becoming (Di Paolo, 2021). Early intervention programs can provide a structured environment that facilitates such interactions, enabling both the infant and the parents to engage in a shared history of development. These shared experiences may contribute to ongoing processes of being/becoming for both parties, as they navigate challenges and explore new possibilities together.
